# Geographical variations in maternal lifestyles during pregnancy associated with congenital heart defects among live births in Shaanxi province, Northwestern China

**DOI:** 10.1038/s41598-020-69788-0

**Published:** 2020-07-31

**Authors:** Yini Liu, Huihui Zhang, Jing Li, Chujun Liang, Yaling Zhao, Fangyao Chen, Duolao Wang, Leilei Pei

**Affiliations:** 1 Department of Epidemiology and Health Statistics, School of Public Health, Xi’an Jiaotong University Health Science Center, Xi’an, 710061 Shaanxi People’s Republic of China; 2 Biostatistics Unit, Department of Clinical Sciences, Liverpool School of Tropical Medicine, Pembroke Place, Liverpool, L3 5QA UK

**Keywords:** Diseases, Risk factors

## Abstract

In this study, we aimed to explore regional differences in maternal lifestyle during pregnancy related to congenital heart defects (CHD) in Shaanxi province, Northwestern China. A large-scale epidemiologic survey of birth defects among infants born during 2010–2013, was conducted in Shaanxi province. Non-spatial and geographic weighted logistic regression models were used for analysis. The spatial model indicated that passive smoking frequency was positively associated with CHD for 43.3% of participants (*P* < 0.05), with the highest OR in North Shaanxi and the lowest in South Shaanxi. Approximately 49.2% of all mothers who ever drink tea were more likely to have an infant with CHD (*P* < 0.05), with the highest OR values observed in North and Central Shaanxi. Additionally, maternal alcohol intake frequency ≥ 1/week was significantly correlated with CHD among about 24.7% of participants (*P* < 0.05), with OR values ranging from 0.738 (Central Shaanxi) to 1.198 (North Shaanxi). The rates of unhealthy maternal lifestyles during pregnancy associated with CHD differed in various areas of the province. The role of geographical variations in these factors may provide some possible clues and basis for tailoring site-specific intervention strategies.

## Introduction

Congenital heart disease (CHD), a serious structural abnormality of the heart or large blood vessels in the intrathoracic, is one of the most common types of birth defects (BD) globally^1,2^. Researches have shown that CHD is a major risk factor for infant mortality associated with BD and can lead to chronic disability, morbidity and increased medical costs^3–5^. It was previously reported that the prevalence of CHD was highest in Asia, second highest in Europe, and lowest in Africa, reflecting significant geographic differences^6–8^. Although environmental and behavioral components play a crucial role in the etiological progression of BD, additional research is still required^9–11^. Over the years, numerous studies exploring risk factors for CHD have confirmed that maternal lifestyle-related factors such as smoking and alcohol intake during pregnancy may be responsible for certain categories of congenital defects^1,12–14^. Because of socio-cultural and geographical reasons, maternal lifestyle-related factors in pregnancy may be different and population-specific in each area. Study results related to associations of maternal lifestyle factors with CHD have not always been consistent across regions^15^. These conflicting results may in part stem from geographic heterogeneity, which would lead to bias in correlation estimates and associated calculations of standard error.

In China, the incidence rate of CHD is the highest among BD, and the surgical treatment of CHD infants is estimated to cost 12 billion Yuan per year^16^. Our previous study found that the Shaanxi province, which accounts for about half of the population in northwest China, is facing more serious BD than other regions of China^17^. Simultaneously, due to extremely unbalanced economic development and huge differences in socio-culture among Shaanxi regions, maternal lifestyle during pregnancy is inevitably disparate across different surveyed areas, and spatial heterogeneity may play an important role in the correlation between the incidence of CHD and maternal lifestyles during pregnancy. Therefore, we hypothesized that the relationship between maternal lifestyle during pregnancy and CHD varied significantly from region to region in Shaanxi. In most previous studies, however, conventional regression models were used to produce average parameters to explore the relationship between maternal lifestyle during pregnancy and CHD over the whole study area, but spatial heterogeneity was neglected^17–20^. In the present study, we used the Geographically Weighted Logistic Regression (GWLR) model to generate local coefficients to account for geographical variations in evaluating the correlation between CHD in infants and maternal lifestyles during pregnancy. GWLR allows for the examination of local changes and provides a map for the visualization of spatial heterogeneity^21^. This geographically weighted regression model was helpful in identifying distinct patterns that appeared in different villages and townships, so that we could clearly visualize differences in maternal lifestyles during pregnancy related to CHD among disparate regions. Thus, the current study aimed to (1) describe the geographic distribution of maternal lifestyles during pregnancy across different regions, (2) and explore the spatially varying relationship between maternal lifestyles during pregnancy and CHD adjusting for other possible confounding factors.

## Results

### Baseline characteristics of the participants

In this study, a total of 1605 villages were chosen within 272 townships of 30 counties in Shaanxi province, and 29,098 live infants were included in the final analysis. Among the infants included in the study, average age was 16.9 ± 11.3 months (range 0–46 months); 54.4% were male; 98.8% were singletons. Among the mothers included in the study, average age was 28.01 ± 4.86 years; average parity was 1.65 ± 0.82. The proportions of study participants from northern, central, and southern Shaanxi, were 25.7%, 53.9% and 20.3%, respectively. The baseline characteristics of the participants across different areas in Shaanxi province are presented in Table 1.

**Table 1:** Baseline characteristics of study population by different areas in Shaanxi province.

Study variable	North Shaanxi n (%)	Central Shaanxi n (%)	South Shaanxi n (%)	*P*
**Socio-demographic factors**				
Fetal gender				
Male	4253 (56.9)	8461 (53.9)	3217 (54.4)	0.378
Female	3223 (43.1)	7233 (46.1)	2698 (45.6)	
Fetal number				
Singleton	7380 (98.6)	15512 (98.8)	5854 (98.9)	< 0.001
Twin and multi-fetal	100 (1.3)	184 (1.2)	65 (1.1)	
Infant parity				
1	3758 (50.2)	9505 (60.5)	3418 (57.7)	< 0.001
2	3287 (43.9)	5672 (36.1)	2289 (38.7)	
≥ 3	393 (5.3)	463 (2.9)	150 (2.5)	
Childbearing age				
18–24	2209 (29.5)	3227 (20.6)	1792 (30.3)	0.008
25–29	3303 (44.2)	6862 (43.7)	2145 (36.2)	
≥ 30	1893 (25.3)	5342 (34.0)	1869 (31.6)	
Mother’s education				
Primary school and below	1640 (21.9)	971 (6.2)	913 (15.4)	0.920
Junior high school	3639 (48.6)	7236 (46.1)	3536 (59.7)	
Senior high school	1154 (15.4)	3616 (23.0)	1042 (17.6)	
College and above	1043 (13.9)	3857 (24.6)	398 (6.7)	
Mother's marriage				
First marriage	6660 (89.0)	6855 (43.7)	5533 (93.5)	0.569
Others	67 (0.9)	163 (1.0)	191 (3.2)	
Household registration				
Urban	1491 (19.9)	1775 (11.3)	410 (6.9)	0.537
Rural	5242 (70.1)	5247 (33.4)	5342 (90.3)	
Household wealth Index				
Poor	2734 (36.5)	5192 (33.1)	1702 (28.8)	0.421
Middle-income	2317 (31.0)	5459 (34.8)	1888 (31.9)	
Rich	2430 (32.5)	5047 (32.2)	2329 (39.3)	
Altitude				
< 500	0	7862 (50.1)	1483 (25.1)	0.244
500–1000	2630 (35.2)	5052 (32.2)	4436 (74.9)	
> 1000	4851 (64.8)	2784 (17.7)	0	
Mother's residence during pregnancy				
Permanent	6897 (92.2)	13631 (86.8)	5113 (86.4)	0.001
Floating	502 (6.7)	1995 (12.7)	784 (13.2)	
Family history of CHD				
No	7388 (98.8)	15549 (99.1)	5875 (99.3)	< 0.001
Yes	59 (0.8)	92 (0.6)	19 (0.3)	
**Maternal lifestyle status during pregnancy**				
Alcohol intake frequency				
No	7442 (99.5)	15541 (99.0)	5740 (97.0)	0.078
< 1/week	12 (0.2)	99 (0.6)	102 (1.7)	
≥ 1/week	19 (0.3)	44 (0.3)	56 (0.9)	
Passive smoking frequency				
No	5080 (67.9)	12438 (79.2)	4710 (79.6)	0.002
< 1/week	427 (5.7)	726 (4.6)	269 (4.5)	
≥ 1/week	1931 (25.8)	2455 (15.6)	838 (14.2)	
Tea consumption				
No	7398 (98.9)	15,424 (98.3)	5519 (93.2)	0.003
Yes	69 (0.9)	248 (1.6)	383 (6.5)	
Coffee consumption				
No	7436 (99.4)	15,564 (99.1)	5823 (98.4)	0.790
Yes	31 (0.4)	110 (0.7)	80 (1.4)	

Values are given as number and proportion of the study population; differences in socio-demographic characteristics between 2009 and 2013 were tested using χ^2^ tests.

The overall prevalence of CHD among the study respondents was 0.760%, with a range from 0 to 4.4% among Shaanxi Province. Notably, the rate of CHD among subjects was higher in South Shaanxi (1.1%) than the corresponding rate in North Shaanxi (0.5%), and Central Shaanxi (0.8%) (Supplementary Fig. S1). The rate of CHD among infants increased markedly in association with increases in fetal number or infant parity (*P* for trend < 0.001). A family history of CHD was associated with increased incidence of CHD among infants (*P* for trend < 0.001). The results obtained showed that the prevalence of CHD increased with maternal age (*P* for trend < 0.01). The rate of CHD was higher in the floating population than in the permanent population (*P* = 0.001). The results obtained showed that maternal lifestyle habits during pregnancy, such as second-hand smoking (*P* for trend < 0.01) and tea consumption (*P* for trend < 0.01), may increase risk for CHD.

The proportion of mothers with a history of passive smoking in pregnancy was highest in North Shaanxi (32.1%), followed by Central Shaanxi (20.8%), and South Shaanxi (20.4%) (Supplementary Fig. S2). With regard to the consumption of alcohol, tea and coffee during pregnancy, South Shaanxi was the area with the highest rate (3.0%, 6.5% and 1.4%, respectively), compared to Central Shaanxi (1.0%, 1.6% and 0.8%, respectively), and North Shaanxi (0.5%, 0.9% and 0.4%, respectively) (Supplementary Fig. S3–S5).

### Non-spatial logistic regression

Table 2 summarized the significant correlations between CHD and maternal lifestyle-related factors using a logistic regression model adjusting for other possible risk factors. Compared with single fetal births, twin and multi-fetal births were associated with a substantial increase in CHD incidence (OR 5.190, 95%CI 2.293–11.750). CHD was more likely to occur in the children of mothers ≥ 30 years of age, compared with children of mothers 18–24 years of age (OR 2.353, 95% CI 1.338–4.140). The rate of CHD was higher in the floating population (OR 1.933, 95% CI 1.261–2.963) than in the permanent population. Participants who had a family history of CHD, were more likely to suffer from CHD (OR 11.250, 95%CI 4.675–27.075). Compared to those living in South Shaanxi, those living in Central Shaanxi and North Shaanxi had decreased risk for CHD (OR 0.440, 95%CI 0.251–0.773 and OR 0.591, 95%CI 0.382–0.916, respectively).

**Table 2:** Non-spatial logistical regression results of CHD.

Study variable	β	Wald χ^2^	*P* value	Adjusted OR	Adjusted OR 95%CI
Constant	− 5.884	60.877	< 0.001	0.003	–
**Socio-demographic factors**					
Fetal gender(ref = Female)					
Male	0.062	0.128	0.720	1.064	0.759–1.492
Fetal number(ref = Singleton)					
Twin and multi-fetal(1 = yes, 0 = no)	1.647	15.603	< 0.001	5.190	2.293–11.750
Infant parity(ref = one)					
2(1 = yes, 0 = no)	− 0.063	0.079	0.779	0.939	0.603–1.460
≥ 3(1 = yes, 0 = no)	0.160	0.136	0.713	1.174	0.500–2.757
Childbearing age(ref = 18–24)					
25–29(1 = yes, 0 = no)	0.383	2.114	0.146	1.466	0.875–2.456
≥ 30(1 = yes, 0 = no)	0.856	8.817	0.003	2.353	1.338–4.140
Mother’s education(ref = Primary school and below)					
Junior high school	0.173	0.412	0.521	1.189	0.701–2.018
Senior high school	0.042	0.015	0.903	1.043	0.529–2.055
College and above	0.555	2.131	0.144	1.741	0.827–3.666
Mother's marriage(ref = Others)					
First marriage	0.524	0.775	0.379	1.689	0.526–5.425
Household registration(ref = Urban)					
Rural	− 0.055	0.043	0.835	0.947	0.566–1.584
Household wealth Index(ref = Poor)					
Middle-income	− 0.032	0.023	0.880	0.968	0.639–1.469
Rich	− 0.104	0.228	0.633	0.902	0.589–1.380
Altitude(ref = less than 500)					
500–1000(1 = yes, 0 = no)	− 0.093	0.175	0.676	0.911	0.590–1.407
> 1000(1 = yes, 0 = no)	− 0.363	1.392	0.238	0.696	0.381–1.271
Mother's residence during pregnancy(ref = Permanent)					
Floating(1 = yes, 0 = no)	0.659	9.131	0.003	1.933	1.261–2.963
Family history of CHD(1 = yes, 0 = no)	2.420	29.180	< 0.001	11.250	4.675–27.075
Area(ref = South Shaanxi)					
Central Shaanxi(1 = yes, 0 = no)	− 0.821	8.163	0.004	0.440	0.251–0.773
North Shaanxi(1 = yes, 0 = no)	− 0.526	5.545	0.019	0.591	0.382–0.916
**Maternal lifestyle status during pregnancy**					
Alcohol intake frequency(ref = no)					
< 1/week(1 = yes, 0 = no)	0.189	0.067	0.796	1.208	0.289–5.057
≥ 1/week(1 = yes, 0 = no)	− 0.138	0.017	0.896	0.871	0.110–6.907
Passive smoking frequency(ref = no)					
< 1/week(1 = yes, 0 = no)	− 0.082	0.037	0.848	0.921	0.399–2.126
≥ 1/week(1 = yes, 0 = no)	0.896	22.439	< 0.001	2.449	1.691–3.548
Tea consumption(1 = yes, 0 = no)	0.678	3.327	0.068	1.971	0.951–4.085
Coffee consumption(1 = yes, 0 = no)	0.073	0.009	0.923	1.076	0.243–4.755

Compared to infants born to mothers without a history of passive smoking during pregnancy, infants born to women with a history of passive smoking during pregnancy had increased risk for CHD (OR 2.449, 95%CI 1.691–3.548). However, other maternal lifestyles during pregnancy, including the frequency of alcohol intake, and consumption of tea and coffee, were not significantly associated with CHD in surveyed areas.

### Multivariate spatial logistic regression

Based on the results of GWLR, we determined that the optimal bandwidth size was 27,875,000 (persons). When the results of global regression model fitting were viewed in combination with the results from GWLR, we concluded that the geographically weighted regression had better fitness. The effects of spatial variation on local estimates for CHD risk factors, as determined using the GWLR model are presented in Table 3.

**Table 3:** Results of the local estimates from the GWLR model of CHD.

Study variable	Minimum		Medium		Maximum		Significant proportion (%)
	β	OR	β	OR	β	OR	
Constant	− 6.026	0.002	− 5.034	0.007	− 2.393	0.091	100
**Maternal lifestyle status during pregnancy**							
Alcohol intake frequency(ref = no)							
< 1/week(1 = yes, 0 = no)	− 0.356	0.700	− 0.013	0.987	0.089	1.093	0
≥ 1/week(1 = yes, 0 = no)	− 0.304	0.738	− 0.168	0.845	0.181	1.198	24.7
Passive smoking frequency(ref = no)							
< 1/week(1 = yes, 0 = no)	− 0.114	0.892	− 0.061	0.941	0.188	1.207	0
≥ 1/week(1 = yes, 0 = no)	0.006	1.006	0.154	1.166	0.464	1.590	43.3
Tea consumption(1 = yes, 0 = no)	0.005	1.005	0.158	1.171	0.222	1.249	49.2
Coffee consumption(1 = yes, 0 = no)	− 0.442	0.643	− 0.025	0.975	0.042	1.043	0

Minimum, Medium, and Maximum denote the minimum, median, and maximum local estimate values, respectively.

β is the estimated effect of the independent variable; OR is adjusted OR.

Significant proportion denotes the proportion of individuals with significant risk factors for CHD.

**Figure 1:**
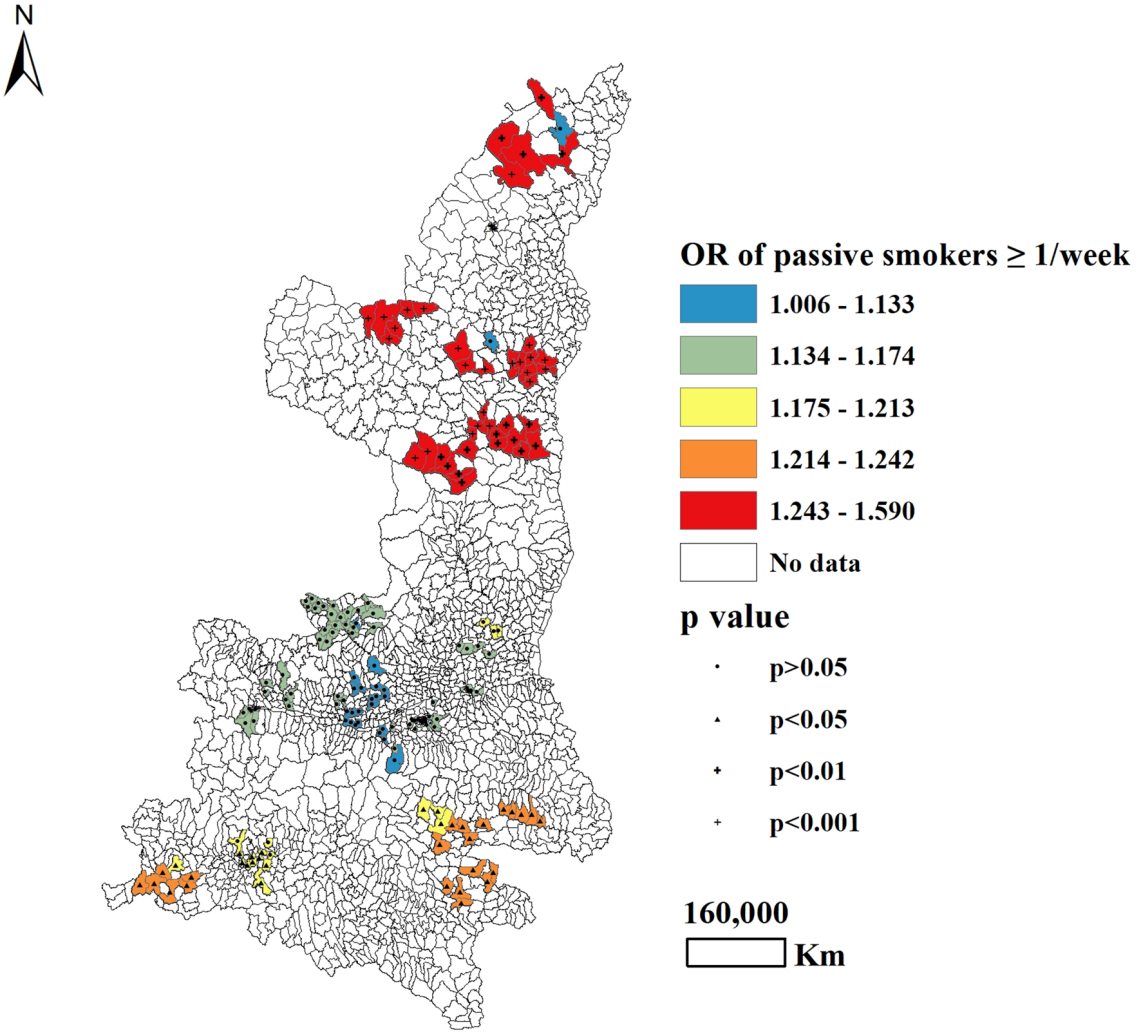
Geographical distribution of the adjusted ORs for frequency of passive smoking, as associated with the prevalence of CHD.

**Figure 2:**
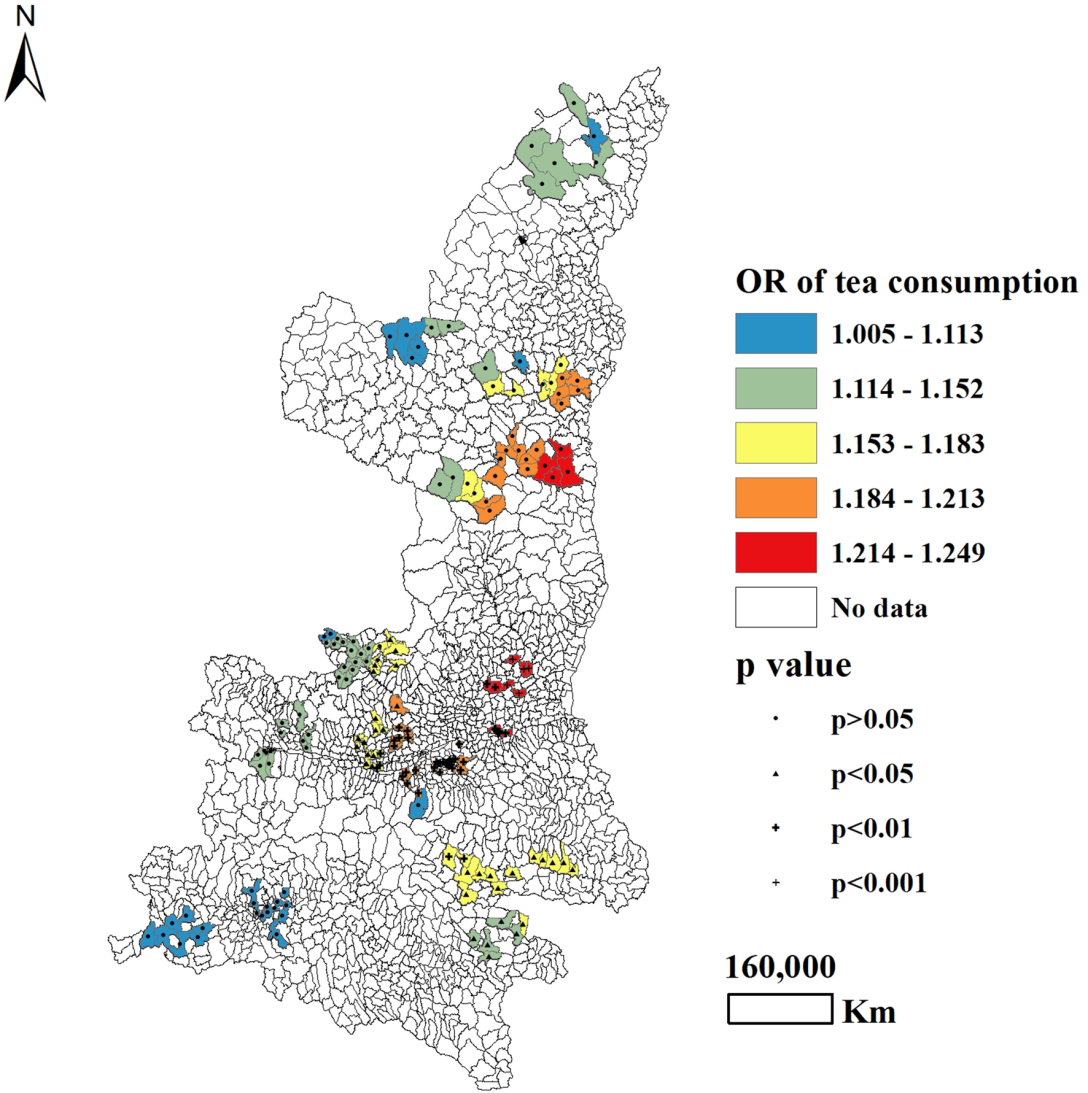
Geographical distribution of the adjusted ORs for tea consumption, as associated with the prevalence of CHD.

**Figure 3:**
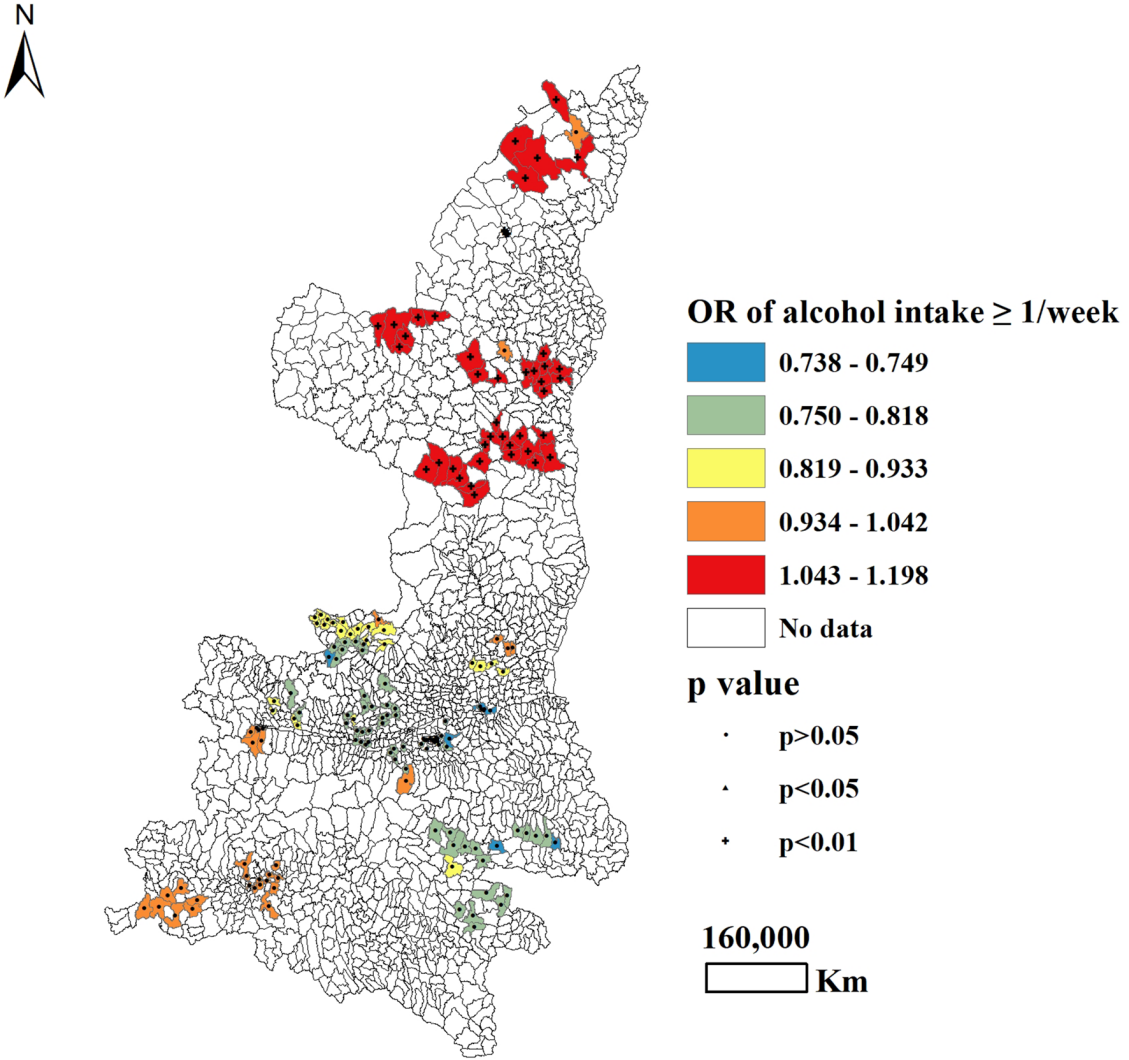
Geographical distribution of the adjusted ORs for the frequency of alcohol intake, as associated with the prevalence of CHD.

With regard to maternal lifestyles during pregnancy, the frequency of passive smoking was found to be positively associated with CHD for 43.3% of participants in South and North Shaanxi province (*P* < 0.05), with the highest OR observed in North Shaanxi and the lowest OR observed in South Shaanxi (Table 3 and Fig. 1). Approximately 49.2% of all mothers who ever drank tea during pregnancy were more likely to have an infant with CHD, compared to mothers who never drank tea during pregnancy (*P* < 0.05). For this trend, OR values were highest in North and Central Shaanxi (Table 3 and Fig. 2). Maternal alcohol intake more than once per week was significantly correlated with CHD occurrence among 24.7% of participants (*P* < 0.05), all of whom lived in North Shaanxi, with adjusted OR values ranging from 0.738 (Central Shaanxi) to 1.198 (North Shaanxi) (Table 3 and Fig. 3).

## Discussion

Using the results from a large-scale cross-sectional survey conducted in Shaanxi province in northwestern China, we explored the geographic variation of some maternal lifestyle-related factors during pregnancy in relation to the risk of CHD after controlling for other baseline characteristics using spatial regression models.

The results of a study conducted previously in China indicated that the rate of passive smoking among pregnant women ranged from 26.6% to 45.3%, which were 17.1% in Shanghai, 34.88% in Shenzhen and 33.96% in Zhengzhou respectively^22^. Similarly, our study showed that the proportion of mothers with a history of passive smoking in pregnancy was 23.6% (range 5.5–57.5%) in Shaanxi, which was highest in North Shaanxi (32.1%). These results showed a significant regional variation in Shaanxi. Simultaneously, the rates of the maternal consumption of alcohol, tea and coffee during pregnancy were higher in South Shaanxi in comparison to other areas of Shaanxi. These findings were consistent with regional distribution of CHD incidence, which showed a decreasing trend from north to south in Shaanxi province.

Global regression analysis revealed that mothers who had a history of passive smoking during pregnancy were more likely to give birth to a baby with CHD. Studies conducted in the UK and the USA reported similar findings^23,24^. The GWLR model showed that the OR for passive smoking during pregnancy was highest in North Shaanxi and lowest in Central Shaanxi. Our survey data indicated that passive smoking was higher in North Shaanxi than in Central Shaanxi or South Shaanxi. Most parts of Central Shaanxi are developed urban areas (e.g., Xi'an), where tobacco control measures have been implemented. In comparison with other areas of Shaanxi, Central Shaanxi has more public places that have become true smoke-free environments. Second-hand smoke may therefore be less of an issue for pregnant women in Central Shaanxi. In contrast, the area surveyed in North Shaanxi comprise small townships and villages, where efforts at tobacco control have not been sufficient to reduce the harmful effects of tobacco. One research study conducted in Shaanxi found that a high proportion of participants, especially less educated mothers in North Shaanxi and South Shaanxi, were exposed to environmental tobacco smoke for more than 15 min on at least 1 day per week^17^. These results remind us that it is extremely urgent to take adequate measures to control tobacco smoking, especially in North Shaanxi. Additional studies will be required to corroborate these findings and to elucidate possible reasons for this geographical variations.

Numerous previous studies have found that tea consumption during pregnancy could adversely affect fetal growth and development^25–27^. In our study, the non-spatial logistic regression models showed no significant association between tea consumption during pregnancy and CHD. However, the GWLR model revealed that tea consumption during pregnancy may increase the risk for CHD in 49.2% of all participants (mostly in Central and South Shaanxi province). When we focus on the map drawn by OR values, it is easy to see that OR values were highest in areas near the eastern part of Shaanxi, with a trend of gradual decline from east to west. On account of the high-altitude landform and warm and humid climate environment in South Shaanxi, the region is suitable for the growth of tea. Local residents therefore consume more tea than inhabitants of other areas. The results of our survey showed that the rate of tea consumption was highest in southeast Shaanxi (6.5%), followed by Central Shaanxi (1.6%), and then by North Shaanxi (0.9%). Household economic level may play a role in tea consumption. The socioeconomic stratification of residents in Shaanxi province has been described in previous studies^17,18^. As a consumer's income increases, he or she is more likely to drink tea^28^. However, more research is needed to unravel the geographic patterns modulating the harmful effects of tea in relation to risk for CHD.

Although the non-global regression results showed no significant correlation between maternal history of alcohol intake and the likelihood of having a child with CHD, the spatial regression model revealed a positive association between maternal history of alcohol intake and the risk of CHD for 24.7% of mothers residing in North Shaanxi. On the map used to visualize these results, the areas with the highest OR values for CHD were mainly distributed in North Shaanxi, followed by South and Central Shaanxi. The relationship between maternal alcohol consumption and CHD has long been studied, with conflicting results. Studies in animal models have identified a significant association between maternal exposure to alcohol during pregnancy and an increased incidence of heart anomalies in offspring^29,30^. Research conducted by Polygenis et al. revealed no effect of maternal alcohol consumption on fetal malformations^31^. A review by Henderson et al. found that only one study reported a significant increase in risk for fetal malformations among mothers who consumed alcohol during pregnancy^32^. Geographical variations may explain, at least in part, the inconsistent results reported for the associations between maternal exposure to alcohol during pregnancy and CHD.

However, several limitations should be considered when interpreting our results. Because this research was based on a cross-sectional study of the population, the causal relationship between maternal lifestyle during pregnancy and CHD in offspring could not be demonstrated. Secondly, due to the limited number of areas sampled (only 30 counties and 175 townships), the variation trends in relevant factors were difficult to isolate. Thirdly, the reported information on maternal lifestyle during pregnancy such as that related to alcohol intake, passive smoking, and consumption of tea or coffee was self-reported by mothers. The duration of time that had elapsed between pregnancy and the maternal self-report may have contributed to recall bias. Therefore, we consciously adopted systematic approaches to maximize power and minimize bias in the study. For example, all questionnaires and procedures were pre-tested, and detailed interviewer guides were developed before the formal survey. During maternal interviews, the standardized questionnaire and detailed supporting material (e.g., calendars) were provided to participants to ensure that complete and accurate responses was obtained. Reporting accuracy was ensured for mothers through highly structured interviews by skilled field staff from Xi’an Jiaotong University Health Science Center and experienced doctors from local maternal and children’s hospitals who were blinded to the study hypotheses. Fourthly, the study subject to other potential confounders such as maternal illness, drug use, genetics, maternal obesity, and environmental factors. Despite these limitations, this study was the first exploration of the geographic varying association of maternal lifestyle factors with CHD in northwestern China, and filled in some of the gaps.

In conclusion, the prevalence of CHD among live births and the rates of the maternal consumption of alcohol, tea and coffee were higher in South Shaanxi, while the proportion of mothers with a history of passive smoking in pregnancy was highest in North Shaanxi. Maternal alcohol consumption and passive smoking during pregnancy may be important risk factors for CHD, especially in North Shaanxi. Tea consumption during pregnancy appeared to increase risk for CHD in North and Central Shaanxi.

## Materials and methods

### Study design and participants

From August to November 2013, a large-scale epidemiologic survey of BD among infants born during 2010–2013, was conducted in Shaanxi province in northwestern China. Because of the imbalance in population density between rural and urban areas, a stratified multi-stage sampling method was used to determine sampling units. While China’s rural areas, counties, townships, and villages have a three-level administrative structure, the administrative structure in urban areas consists of districts, streets, and communities. Twenty counties and ten districts were randomly selected for inclusion in the study. Six townships in each county were chosen, and 6 villages in each selected township were randomly selected. In urban areas, 3 streets in the chosen district and 6 communities on the chosen street were selected randomly. In the end, a completely random sampling method was adopted to select 30 live births and their mothers for each selected village and 60 live births and their mothers for each selected community. The response rate was 92.7%, and a total of 29,098 live babies were ultimately enrolled, representing 9.00% of the province’s total infant population^18^.

### Data collection

Prior to the formal survey, all questionnaires and procedures were pre-tested, and detailed interviewer guides were developed. The final questionnaire had four parts: birth defects questionnaire, family questionnaire, reproductive history questionnaire, and family history questionnaire. In the survey, all the major data collected was reported by mothers with live births. After written informed consent was obtained, face-to-face interviews were performed to extract data on sociodemographic characteristics and maternal lifestyle during pregnancy^18^.

During the investigation, ten survey teams were established for field data collection in the areas surveyed, with each group consisting of 10–12 investigators, a supervisor from Xi'an Jiaotong University Health Science Center, and a pediatrician from the local maternal and child health hospital. Before the start of the survey, all investigators were trained via lectures and field exercises to standardize implementation of the questionnaire. The supervisors were required to report any questionnaires with errors and/or incomplete information. The doctors in each team helped to collect information on BD. The study was strongly supported by local hospitals, administrative health services, and the Shaanxi Provincial Ministry of Health. All data collection occurred at local village clinics and community health service centers. The study was reviewed and approved by the Ethics Committee of Xi'an Jiaotong University Health Science Center, and all methods involving humans were carried out in accordance with relevant guidelines (Declaration of Helsinki).

The diagnosis of CHD was a multistage process. As a first step, infants were screened for CHD by a doctor from the local maternal and child health hospital. Isolated patent foramen ovale and patent ductus arteriosus among neonates < 28 days of age were considered to be normal neonatal findings and excluded from the study. As soon as infants were found to have CHD, their mothers were interviewed. The attending physician reviewed the medical record and obtained information pertaining to diagnosis time, hospital, and type of CHD. For new cases of suspected CHD, a special neonatal echocardiogram and electrocardiogram were required for a definitive diagnosis at the First Affiliated Hospital of Xi'an Jiaotong University^18^.

In this study, the vector map of Shaanxi province was obtained through the “Cropping” function of ArcMap, from the Chinese vector map. Meanwhile, geographic information for each township/street in Shaanxi province was acquired from the Geographic Information Comprehensive Service Website (https://www.tianditu.com/). The information obtained included geographic coordinates, boundaries, and altitude. The basic geographic units in this study were townships and streets. Geographic location was defined as the geographic coordinates (i.e., latitude/longitude) for the township/street center where the participant lived.

### Study variables

In the study, maternal lifestyle during pregnancy included the frequency of alcohol intake (no, < 1/week, ≥ 1/week), frequency of passive smoking (no, < 1/week, ≥ 1/week), and consumption of tea and coffee during pregnancy (yes, no). Frequency of passive smoking was defined as the number of times that the respondent passively inhaled smoke for > 15 min per day. Before analysis, the frequency of alcohol intake and passive smoking were divided into two dummy variables, respectively: < 1/week (1 = yes, 0 = no), ≥ 1/week (1 = yes, 0 = no), with no as a reference group.

Other socio-demographic factors included fetal gender (male, female), fetal number (singleton, twin and multi-fetal), infant parity (1, 2, or ≥ 3), childbearing age (18–24, 25–29, ≥ 30 years), mother’s education (primary school and below, junior high school, senior high school, college and above), mother's marriage (first marriage, others), household registration (urban, rural), Household wealth Index (poor, middle-income, rich), altitude of residence (< 500, 500–1000, > 1000), mother's residence during pregnancy (floating, permanent), family history of CHD (no , yes), and area (South Shaanxi, Central Shaanxi, North Shaanxi). Household wealth index based on the principal component analysis was used to assess the household economic level of the participants. The first principal component representing family economic level was divided into thirds: low, medium, and high (poor, middle-income, rich, respectively). Any CHD were considered unique outcome variables in the multivariate analysis.

### Statistical analysis

The analysis procedure is shown in detail below. The geographic distribution of maternal lifestyle during pregnancy were shown on a map using ArcGIS 10.0 software (Environmental Systems Research Institute, Inc., Redlands, CA, US). Non-spatial logistic regression analysis was performed to explore the association between maternal lifestyle during pregnancy and CHD controlling for other confounders by means of IBM SPSS Statistics 25. Then, all explanatory variables were entered into the GWLR model. To confirm geographic discrepancy in the relationship between maternal lifestyle during pregnancy and CHD after adjusting for other factors, local average estimates (OR) and *P* values for each individual were displayed on a map using ArcGIS 10.0 software. The GWLR formula is as follows^33^:

$$ \log \left( {\frac{{P\left( {y_{i} = 1} \right)}}{{1 - P\left( {y_{i} = 1} \right)}}} \right) = \beta_{0i} \left( {u_{i} ,v_{i} } \right) + \mathop \sum \limits_{j = 1}^{k} \left( {\beta_{ij} \left( {u_{i} ,v_{i} } \right)x_{ij} } \right) $$

The equation assumes *y*_*i*_ is any CHD for each individual i, *x*_*ij*_ is a set of independent variables (*j* = 1, …, *k*) for individual i, (*u*_*i*_, *v*_*i*_) is the x–y coordinates of individual i; *β*_*ij*_ is the estimated effect of independent variable j for individual i.

The GWLR model was estimated with the iterative reweighted least squares method using GWR 4.0 software (https://geodacenter.asu.edu/software/downloads/gwr_downloads). For modeling, a GWLR equation was estimated for each township/street based on the observations for nearby townships/streets. A distance-based weighting scheme was used to allocate weights to each township/street. The kernel type and function for geographic weighting to estimate local coefficients for each township/street and bandwidth size was adaptive bisquare. The Corrected Akaike Information Criterion (AICc) was used for the golden section search method to select bandwidth in adaptive kernels. The variability of each coefficient in geographic space was tested by comparing different^34^. In the GWLR model, exponentiation of the explanatory variable coefficients was ultimately calculated to acquire the OR corresponding to the unit change in the variable^35^.

## Supplementary information

Supplementary information 1.

Supplementary figure 1.

Supplementary figure 2.

Supplementary figure 3.

Supplementary figure 4.

Supplementary figure 5.

Supplementary information 2.

Supplementary information 3.

## References

[1] Hoffman JI, Kaplan S (2002). The incidence of congenital heart disease. J. Am. Coll. Cardiol..

[2] Mendis S, Puska P, Norrving B (2011). Global Atlas on Cardiovascular Disease Prevention and Control.

[3] Yang Q (2006). Racial differences in infant mortality attributable to birth defects in the United States, 1989–2002. Birth Defects Res. A Clin. Mol. Teratol..

[4] Centers for Disease Control and Prevention (CDC) (2007). Hospital stays, hospital charges, and in-hospital deaths among infants with selected birth defects–United States, 2003. MMWR Morb. Mortal. Wkly. Rep..

[5] Waitzman NJ, Romano PS, Scheffler RM (1994). Estimates of the economic costs of birth defects. Inquiry.

[6] Nelson JS, Strassle PD (2018). Regional differences in right versus left congenital heart disease diagnoses in neonates in the United States. Birth Defects Res..

[7] Ma LG (2018). Spatial pattern and variations in the prevalence of congenital heart disease in children aged 4–18 years in the Qinghai-Tibetan Plateau. Sci. Total Environ..

[8] Linde DVD (2011). Birth prevalence of congenital heart disease worldwide: a systematic review and meta-analysis. J. Am. Coll. Cardiol..

[9] Bower, C., Rudy, E., Callaghan, A., Quick, J., Cosgrove, P. Report of the Birth Defects Registry of Western Australia 1980–2009. King Edward Memorial Hospital: Women and Newborn Health Service Report.

[10] Vaktskjold A, Talykova LV, Nieboer E (2011). Congenital anomalies in newborns to women employed in jobs with frequent exposure to organic solvents–a register-based prospective study. BMC Pregnancy Childbirth.

[11] Luo YL, Cheng YL, Gao XH, Tan SQ, Li JM, Wang W (2013). Maternal age, parity and isolated birth defects: a population-based case-control study in Shenzhen, China. PLoS ONE.

[12] Pate SS, Burns TL (2013). Nongenetic risk factors and congenital heart defects. Pediatr. Cardiol..

[13] Dilber D, Malcić I (2010). Spectrum of congenital heart defects in Croatia. Eur. J. Pediatr..

[14] Easton JF, Stephens CR, Angelova M (2014). Risk factors and prediction of very short term versus short/intermediate term post-stroke mortality: a data mining approach. Comput. Biol. Med..

[15] Liu Y (2019). Global birth prevalence of congenital heart defects 1970–2017: updated systematic review and meta-analysis of 260 studies. Int. J. Epidemiol..

[16] Song X (2012). Depression and its influencing factors among mothers of children with birth defects in China. Matern. Child Health J..

[17] Pei LL, Kang YJ, Cheng Y, Yan H (2015). The Association of Maternal Lifestyle with Birth Defects in Shaanxi Province, Northwest China. PLoS ONE.

[18] Pei LL, Kang YJ, Zhao YL, Yan H (2017). Prevalence and risk factors of congenital heart defects among live births: a population-based cross-sectional survey in Shaanxi province, Northwest China. BMC Pediatr..

[19] Li MM (2019). Association between congenital heart disease and maternal disease in early pregnancy in women of childbearing age. Zhonghua Liu Xing Bing Xue Za Zhi.

[20] Guo LQ (2019). A Matched case-control study on the association between colds, depressive symptoms during pregnancy and congenital heart disease in Northwestern China. Sci. Rep..

[21] Mayfield HJ (2018). Use of geographically weighted logistic regression to quantify spatial variation in the environmental and sociodemographic drivers of leptospirosis in Fiji: a modelling study. Lancet Planet Health.

[22] Xu H (2020). Status of passive maternal smoking during pregnancy and its impact on pregnancy outcomes in 2,241 pregnant women. PPM.

[23] The information center. The Information Centre Statistics on smoking: England 2006. *National Health Service*. Preprint at https://smokefreeengland.co.uk/files/statistics-on-smoking—england-2006.pdf (2006).

[24] Centers for Disease Control and Prevention. PRAMS and Smoking. *Pregnancy Risk Assessment Monitoring System (PRAMS).* Preprint at https://www.cdc.gov/prams/tobaccoandprams.htm.

[25] Aldridge A, Aranda JV, Neims AH (1979). Caffeine metabolism in the newborn. Clin. Pharmacol. Ther..

[26] Arnaud MJ, Bracco I, Sauvageat JL, Clerc MF (1983). Placental transfer of the major caffeine metabolite in the rat using 6-amino-5[N-formylmethylamino]1,3[Me-14C]-dimethyluracil administered orally or intravenously to the pregnant rat. Toxicol. Lett..

[27] Okubo H, Miyake Y, Tanaka K, Sasaki S, Hirota Y (2015). Maternal total caffeine intake, mainly from Japanese and Chinese tea, during pregnancy was associated with risk of preterm birth: the Osaka Maternal and Child Health Study. Nutr. Res..

[28] Guan X, Yang JF, Xie XY, Lin LQ (2011). Research on the status of Chinese tea consumption and factors of tea consumer’s behavior. J. Tea Sci..

[29] Daft PA, Johnston MC, Sulik KK (1986). Abnormal heart and great vessel development following acute ethanol exposure in mice. Teratology.

[30] Beauchemin RR, Gartner LP, Provenza DV (1984). Alcohol induced cardiac malformations in the rat. Anat. Anz..

[31] Polygenis D (1998). Moderate alcohol consumption during pregnancy and the incidence of fetal malformations: a meta-analysis. Neurotoxicol. Teratol..

[32] Henderson J, Gray R, Brocklehurst P (2007). Systematic review of effects of low-moderate prenatal alcohol exposure on pregnancy outcome. BJOG.

[33] Yang TC, Matthews SA (2012). Understanding the non-stationary associations between distrust of the health care system, health conditions, and self-rated health in the elderly: a geographically weighted regression approach. Health Place.

[34] Nakaya T, Fotheringham AS, Brunsdon C, Charlton M (2005). Geographically weighted Poisson regression for disease association mapping. Stat. Med..

[35] Zhou YB (2015). Geographical variations in risk factors associated with HIV infection among drug users in a prefecture in Southwest China. Infect. Dis. Poverty.

