# Supplementary material for: Geographical variations in maternal lifestyles during pregnancy associated with congenital heart defects among live births in Shaanxi province, Northwestern China
Source: Sci Rep. 2020 Jul 31;10:12958. doi: 10.1038/s41598-020-69788-0 (PMC7395152; doi:10.1038/s41598-020-69788-0)
Supplement: Supplementary file 1 — Supplementary information 1. [file 41598_2020_69788_MOESM1_ESM.docx]

**Geographical variations in maternal lifestyles during pregnancy associated with congenital heart defects among live births in Shaanxi province, Northwestern China**

Yini Liu^1^, Huihui Zhang^1^, Jing Li^1^, Chujun Liang^1^, Yaling, Zhao^1^, Fangyao Chen^1^, Duolao Wang^2^, Leilei Pei^1^*

^1^ Department of Epidemiology and Health Statistics, School of Public Health, Xi’an Jiaotong University Health Science Center, Xi’an, Shaanxi 710061, P.R. China

^2^ Biostatistics Unit, Department of Clinical Sciences, Liverpool School of Tropical Medicine, Pembroke Place, Liverpool, L3 5QA, UK.

*Corresponding author: Leilei Pei, Department of Epidemiology and Health Statistics, School of Public Health, Xi’an Jiaotong University Health Science Center, Xi’an, Shaanxi 710061, P.R. China. Tel: +86 29 8265 5104. Fax: +86 29 8265 5387. Email: pll_paper@126.com

**Supplementary information**

**Fig. S1** Geographical distribution of the rate for CHD.

**Fig. S2** Geographical distribution of the rate for maternal history of passive smoking during pregnancy.

**Fig. S3** Geographical distribution of the rate for maternal history of alcohol during pregnancy.

**Fig. S4** Geographical distribution of the rate for maternal tea consumption during pregnancy.

**Fig. S5** Geographical distribution of the rate for maternal coffee consumption during pregnancy.

Informed consent.

The questionnaire information.
