# Supplementary figures and images for: Geographical variations in maternal lifestyles during pregnancy associated with congenital heart defects among live births in Shaanxi province, Northwestern China
Source: Sci Rep. 2020 Jul 31;10:12958. doi: 10.1038/s41598-020-69788-0 (PMC7395152; doi:10.1038/s41598-020-69788-0)

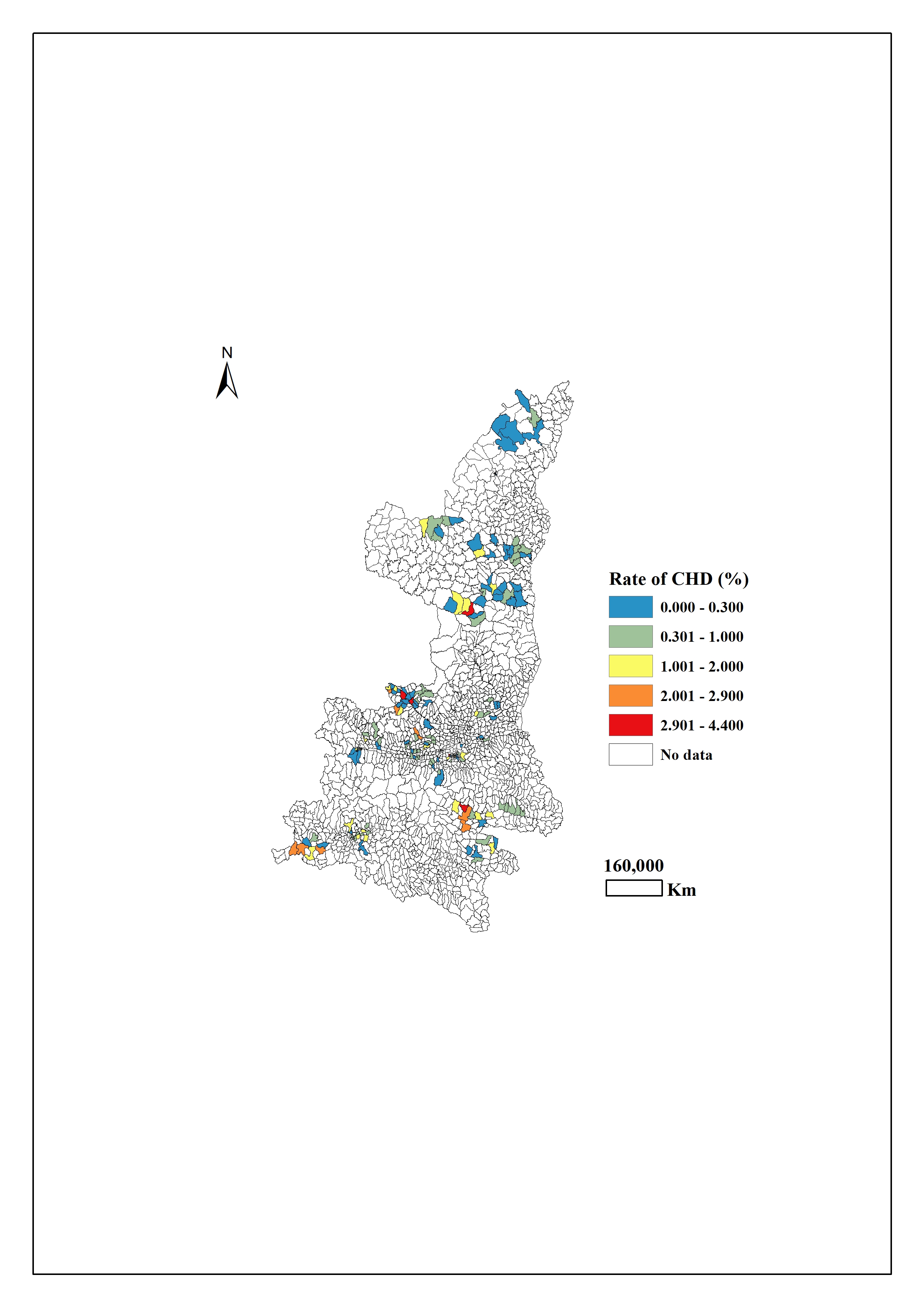

Supplement: Supplementary file 2 — Supplementary figure 1. [file 41598_2020_69788_MOESM2_ESM.jpg]

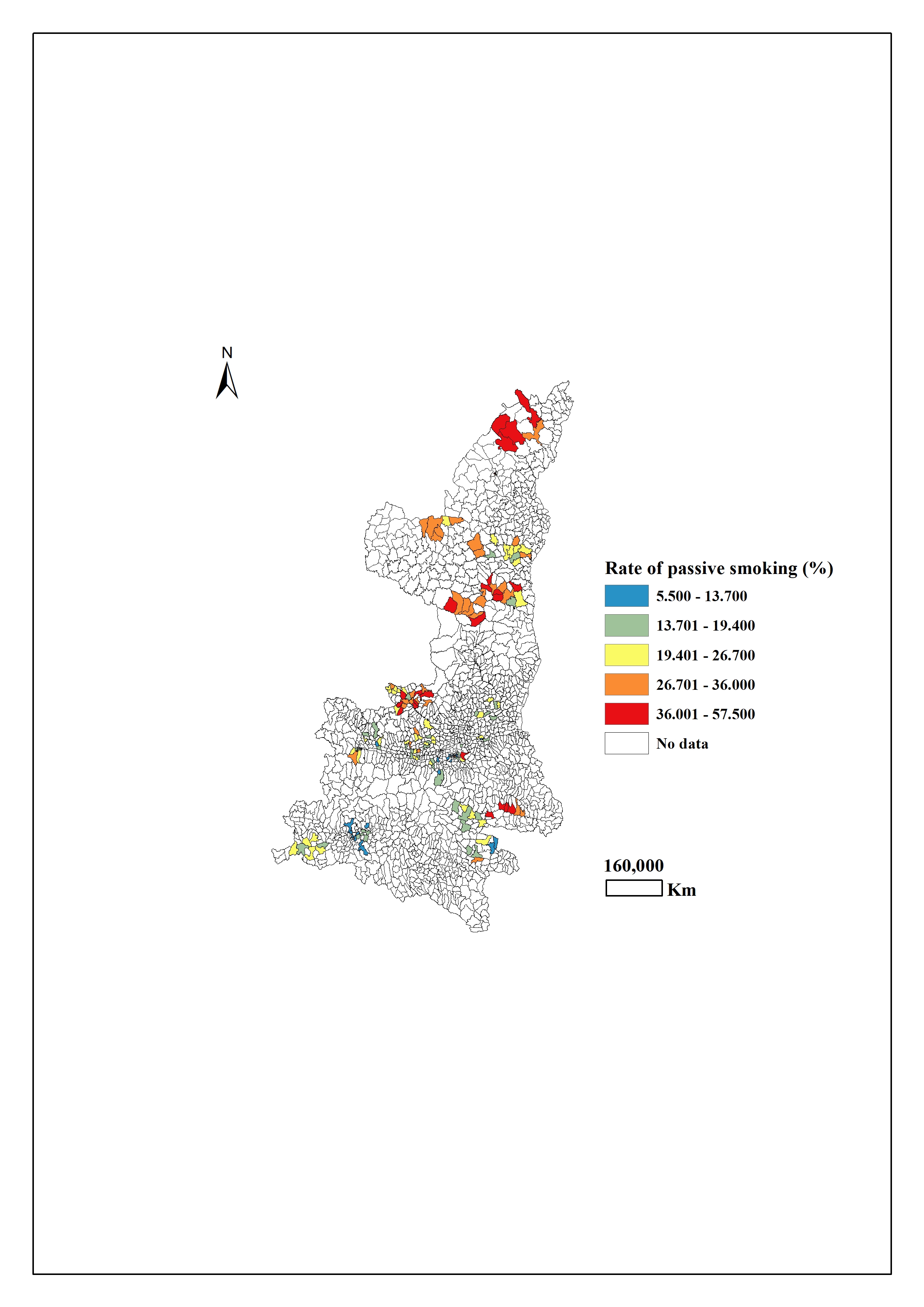

Supplement: Supplementary file 3 — Supplementary figure 2. [file 41598_2020_69788_MOESM3_ESM.jpg]

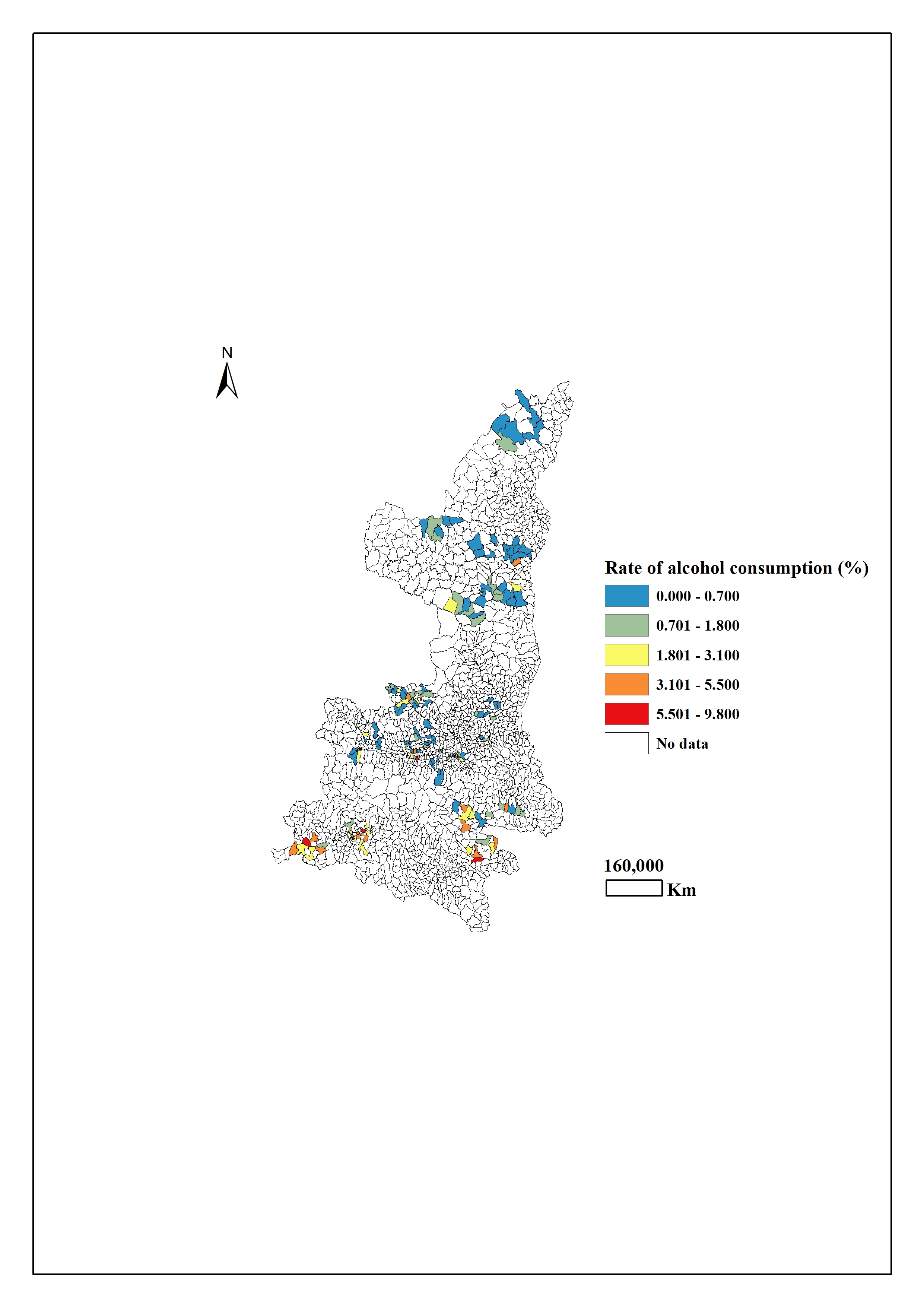

Supplement: Supplementary file 4 — Supplementary figure 3. [file 41598_2020_69788_MOESM4_ESM.jpg]

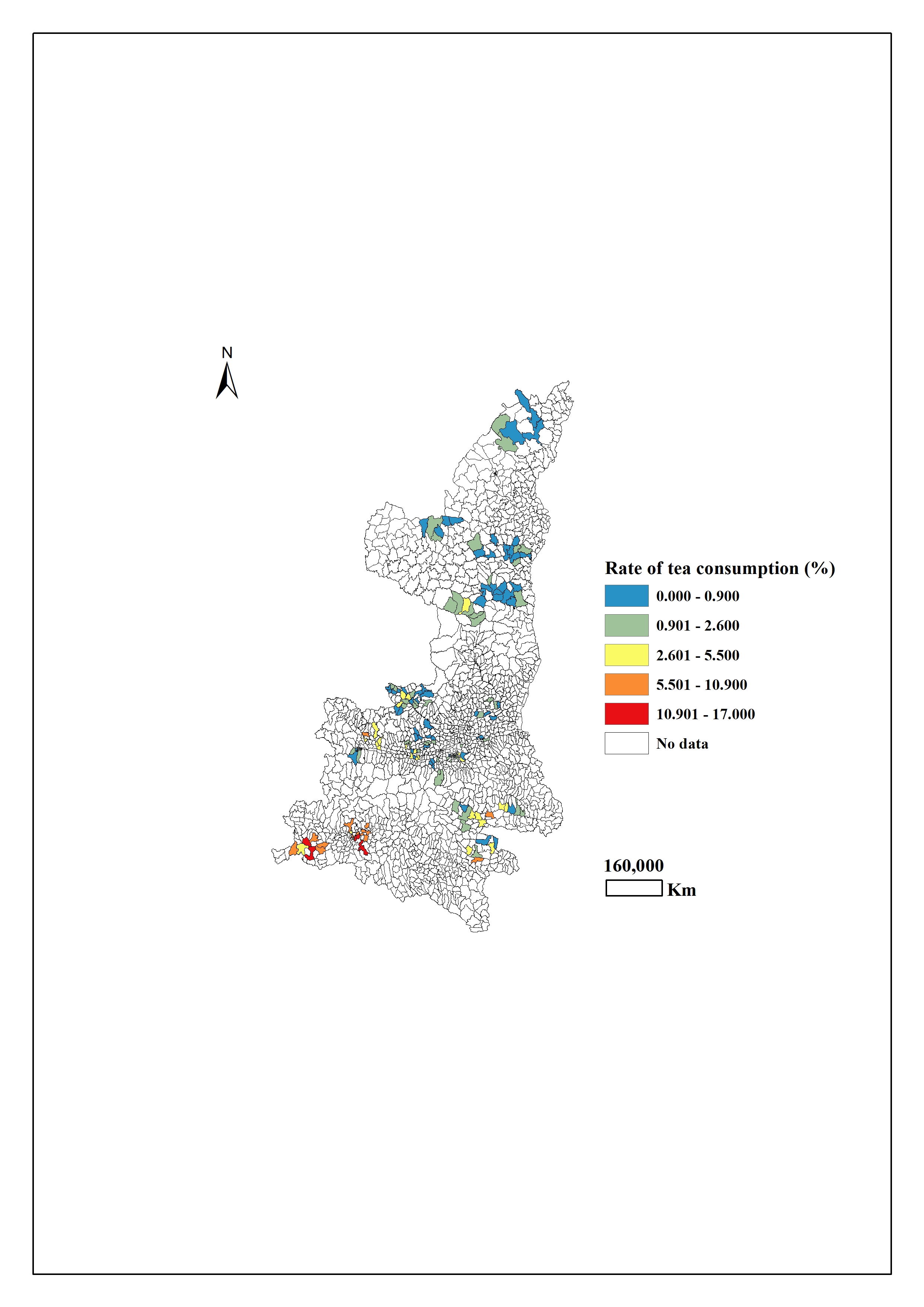

Supplement: Supplementary file 5 — Supplementary figure 4. [file 41598_2020_69788_MOESM5_ESM.jpg]

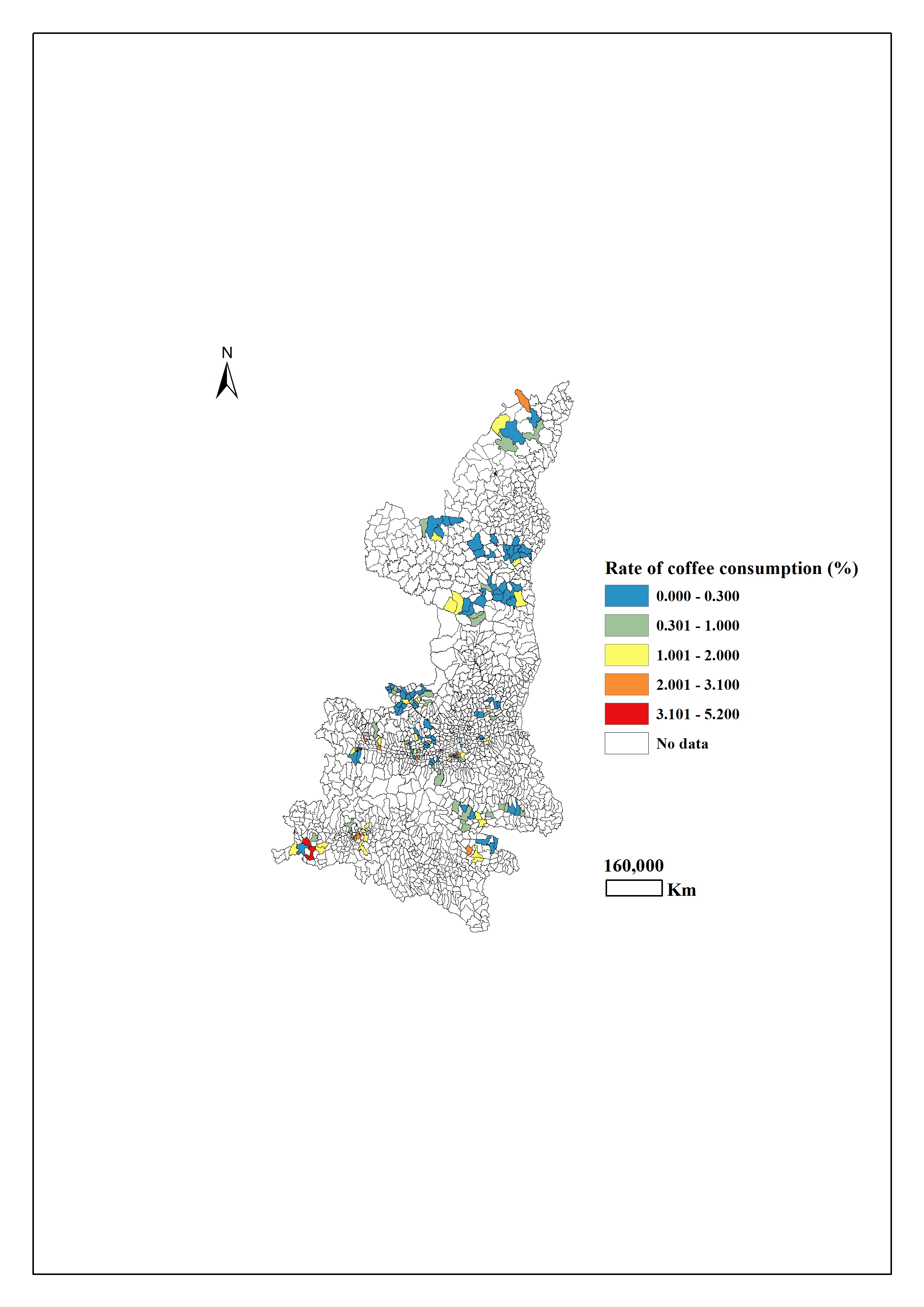

Supplement: Supplementary file 6 — Supplementary figure 5. [file 41598_2020_69788_MOESM6_ESM.jpg]
