# Supplementary material for: Geographical variations in maternal lifestyles during pregnancy associated with congenital heart defects among live births in Shaanxi province, Northwestern China
Source: Sci Rep. 2020 Jul 31;10:12958. doi: 10.1038/s41598-020-69788-0 (PMC7395152; doi:10.1038/s41598-020-69788-0)
Supplement: Supplementary file 7 — Supplementary information 2. [file 41598_2020_69788_MOESM7_ESM.doc]

**Informed consent form**

Hello:

We are investigators from Xi 'an Jiaotong University Health Science Center. The purpose of this study is to understand the occurrence of congenital heart disease and the exposure of related risk factors in our province in the last three years.

If you are willing to join in the project, you need to agree to the following:

1) I will cooperate with the investigator to complete relevant questionnaires

2) For participants with suspected CHD, a specialized neonatal echocardiography and electrocardiography was conducted at the first affiliated hospital of Xi’an Jiaotong University Health Science Center.

We will ensure that all information is confidential and will only be used for this program. The individual information will not be disclosed to anyone without your consent.

If you meet the following conditions, you will be able to participate in the program:

1) You are a permanent resident (registered permanent residence)

2) You were pregnant between 2010 and 2013

You can knowledge research progress of this study at any time. If you have any questions of this study or the rights interests of participants in this study, you can contact the project leader at 029-82655104-207.

I have carefully read and understand all of the above information. In addition, the investigator have answered my questions about project.

**Agree to participate □ Disagree to participate □**

Signature of participants：_______________ Signature of investigators：___________________

Date：201＿/＿ ＿/＿ ＿ Date：201＿/＿ ＿/＿ ＿

…………………………………………………………………………………………………………………………………

**知情同意书**

您好：

我们是来自西安交通大学医学院的调查员，本次研究的目的在于了解我省最近三年出生缺陷（特别是先天性心脏病）的发生情况及相关危险因素的暴露情况。

如果您符合项目的要求，您需要同意以下几项内容：

1. 配合调查员完成相关问卷的填写
2. 必要时将孩子转送上级医院进行心脏超声检查

我们对您所回答的问题和我们收集的有关您孩子的信息保密，并保证这些信息只为本项目使用，没有征得您的同意这些信息不会泄露给任何人。

如果您符合以下条件，您将能够参加本项目：

1）您为本地常驻人员（本地户口）

2）您曾经在2010-2013年间怀孕

**同意参加本项目 □ 不同意参加本项目 □**

受访者签名或手印：_______________ 研究者签名：___________________

日 期：201＿/＿ ＿/＿ ＿ 日 期：201＿/＿ ＿/＿ ＿
